# A transcriptional program associated with cell cycle regulation predominates in the anti-inflammatory effects of CX-5461 in macrophage

**DOI:** 10.3389/fphar.2022.926317

**Published:** 2022-10-26

**Authors:** Jie Wang, Zhijian Zheng, Xiaopei Cui, Chaochao Dai, Jiaxin Li, Qunye Zhang, Mei Cheng, Fan Jiang

**Affiliations:** ^1^ Key Laboratory of Cardiovascular Proteomics of Shandong Province and Department of Geriatrics, Qilu Hospital, Cheeloo College of Medicine, Shandong University, Jinan, Shandong, China; ^2^ Key Laboratory of Cardiovascular Remodeling and Function Research (Chinese Ministry of Education and Chinese National Health Commission), Cheeloo College of Medicine, Qilu Hospital, Shandong University, Jinan, Shandong, China; ^3^ Department of Cardiology, The First Affiliated Hospital of Shandong First Medical University & Shandong Provincial Qianfoshan Hospital, Shandong First Medical University, Jinan, Shandong, China

**Keywords:** RNA polymerase I inhibitor, CX-5461, inflammation, macrophage, transcriptome sequencing, cell cycle, systems biology

## Abstract

CX-5461, a novel selective RNA polymerase I inhibitor, shows potential anti-inflammatory and immunosuppressive activities. However, the molecular mechanisms underlying the inhibitory effects of CX-5461 on macrophage-mediated inflammation remain to be clarified. In the present study, we attempted to identify the systemic biological processes which were modulated by CX-5461 in inflammatory macrophages. Primary peritoneal macrophages were isolated from normal Sprague Dawley rats, and primed with lipopolysaccharide or interferon-γ. Genome-wide RNA sequencing was performed. Gene Ontology and Kyoto Encyclopedia of Genes and Genomes databases were used for gene functional annotations. Enrichment analysis was conducted using the ClusterProfiler package of R software. We found that CX-5461 principally induced a molecular signature related to cell cycle inhibition in primed macrophages, featuring downregulation of genes encoding cell cycle mediators and concomitant upregulation of cell cycle inhibitors. At the same concentration, however, CX-5461 did not induce a systemic anti-inflammatory transcriptional program, although some inflammatory genes such as IL-1β and gp91phox NADPH oxidase were downregulated by CX-5461. Our data further highlighted a central role of p53 in orchestrating the molecular networks that were responsive to CX-5461 treatment. In conclusion, our study suggested that limiting cell proliferation predominated in the inhibitory effects of CX-5461 on macrophage-mediated inflammation.

## 1 Introduction

CX-5461 is a novel selective RNA polymerase I (Pol I) inhibitor which exhibits promising therapeutic efficacies in treatment of certain blood and solid tumors (Drygin et al., 2011; Haddach et al., 2012; Ferreira et al., 2020), with favorable tolerance and safety profiles in human subjects (Khot et al., 2019). Pol I mediates the synthesis of ribosomal RNA, while inhibiting Pol I activity in mammalian cells induces a distinct cellular stress response termed nucleolar stress response, which culminates in stabilization and/or activation of the p53 pathway (Boulon et al., 2010; Bywater et al., 2013; Yang et al., 2018). Specifically, CX-5461 triggers a non-canonical DNA damage response (DDR), featuring activation of the ataxia telangiectasia mutated (ATM)/ATM and Rad3-related (ATR) pathway upstream of p53 (Negi and Brown, 2015; Quin et al., 2016; Ye et al., 2017; Pang et al., 2021). Experimental evidence from our and other laboratories implies that the anti-proliferative effect of CX-5461 in p53-intact cells is mainly mediated by stimulation of the DDR-p53 axis, but not directly attributable to inhibition of ribosome biogenesis (Bywater et al., 2012; Tsai and Pederson, 2014; Quin et al., 2016; Ye et al., 2017; Sanij et al., 2020; Pang et al., 2021). However, the molecular events induced by CX-5461 in the cell leading to ATM/ATR activation is still under debate (Drygin et al., 2011; Quin et al., 2016; Xu et al., 2017; Bruno et al., 2020; Mars et al., 2020).

In addition to its anti-tumor activities, our previous studies have also revealed that CX-5461 has unique pharmacological effects on pathologic vascular remodeling and the accompanying vascular inflammation. For example, CX-5461 treatment effectively suppressed the arterial remodeling induced by orthotopic aortic transplantation and experimental pulmonary arterial hypertension (Dai et al., 2018; Xu et al., 2021); in these experiments, CX-5461 significantly reduced the number of infiltrating adventitial macrophages. *In vitro*, CX-5461 inhibited macrophage proliferation, maturation, and lipopolysaccharide (LPS)-induced expression of pro-inflammatory cytokines (Dai et al., 2018). Because infiltration and activation of adventitial macrophages have crucial roles in promoting the development of vascular inflammation and remodeling (Wang et al., 2018), our results suggest that the modulating effects of CX-5461 on macrophage functions may have a major contribution to its vascular protective actions.

Consistent with our experimental results, there is evidence showing that p53 activation exhibits anti-inflammatory properties under various pathologic conditions [reviewed recently in (Cui et al., 2021)]. In macrophages, p53 may produce anti-inflammatory effects by downregulating the expression of STAT-1 (signal transducer and activator of transcription 1) (Zheng et al., 2005). In addition, p53 may possibly antagonize the pro-inflammatory signaling events downstream of interleukin (IL)-1β receptor, tumor necrosis factor (TNF)-α receptor, and Toll-like receptors, by limiting functions of the master transcription factor NF-κB (nuclear factor-κB) (Wadgaonkar et al., 1999; Webster and Perkins, 1999). It has been well established that pro-inflammatory activation of macrophages has pivotal roles in orchestrating the development of inflammation, thereby making significant contributions to the pathogenesis of most acute and chronic inflammatory diseases, such as infectious sepsis, severe acute respiratory syndromes, autoimmune diseases, allergies, cardiovascular diseases, metabolic syndromes, and cancer (Austermann et al., 2022). At present, however, the molecular mechanisms underlying the inhibitory effects of CX-5461 on macrophage-mediated inflammation remain to be clarified. Based on our previous observations from cell functional studies, here we attempt to identify the systemic biological processes which are modulated by CX-5461 in LPS- and interferon (IFN)-γ-primed macrophages, using contemporary genome-wide RNA sequencing technology. These data may provide a molecular framework for understanding the mechanisms by which CX-5461 suppresses macrophage-mediated inflammation.

## 2 Materials and methods

### 2.1 Purification and culture of peritoneal macrophages

Normal adult Sprague Dawley rats (8–10 weeks of age) were purchased from Vital River Laboratory (Beijing, China). The use of experimental animals was approved by the institutional Animal Ethics Committee of Qilu Hospital of Shandong University. Animals were handled in accordance with the Guideline for the Care and Use of Laboratory Animals (NIH, United States). Peritoneal macrophages were elicited as described (Liu et al., 2016). For each rat, 5 ml of 3% thioglycollate broth (from Sigma, St. Louis, MO, United States) was injected into the peritoneal cavity. After 72 h, the animal was euthanized by intraperitoneal injection of an overdose of pentobarbital sodium. The peritoneal cavity was flushed with 10 ml of PBS solution containing 1 mM EDTA (pH 7.5). The solution was withdrawn using a syringe with 18G needle, and cells were harvested by centrifugation. Cells were resuspended in DMEM medium supplemented with 10% FBS and antibiotics (all from Thermo Fisher, Waltham, MA, United States), and seeded in 6-well plates. After 2 h of incubation, non-adherent cells were removed by gently washing the wells. Cells were used for experimentation when ∼70% of confluence was reached.

### 2.2 Cell treatments

CX-5461 (purchased from ApexBio Technology LLC, Houston, TX, United States) was initially dissolved in DMSO, and diluted in the culture medium to the final concentration of 1 μM (treatment duration 24 h). DMSO was used as vehicle control. LPS was purchased from Solarbio (Beijing, China) and used at the final concentration of 1 μg/ml. IFN-γ was from Proteintech (Wuhan, Hubei Province, China) and used at 150 U/ml.

### 2.3 Sample preparation

The RNA processing and sequencing services were provided by LC-Bio Technology (Hangzhou, Zhejiang Province, China). Total RNA was extracted using Trizol reagent (Thermo Fisher) following the manufacturer’s instructions. The RNA quantity and purity were analyzed using Bioanalyzer 2100 and RNA 6000 Nano LabChip Kit (from Agilent, Santa Clara, CA, United States). RNA samples with RIN number > 7.0 were used for library construction. Purified mRNA was obtained from 5 μg of total RNA using Dynabeads Oligo (dT) (Thermo Fisher) with two rounds of purification; the mRNA was then fragmented using divalent cations (Magnesium RNA Fragmentation Module from New England Biolabs, Ipswich, MA, United States) under 94°C for 5 min.

### 2.4 RNA sequencing and raw data processing

The cleaved mRNAs were reverse-transcribed to cDNAs using SuperScript II Reverse Transcriptase (Thermo Fisher), which were used as templates to synthesize U-labeled second-strand DNAs with *E. coli* DNA polymerase I, RNase H (all from New England Biolabs) and dUTP (Thermo Fisher). After ligation with Dual-index adapters, the DNA library was treated with heat-labile UDG enzyme (New England Biolabs) to remove the U-labeled second strands, and amplified by PCR. The average insert size of the final cDNA library was 300 bp ± 50 bp. Paired-end sequencing (2 bp × 150 bp) was performed using a NovaSeq 6000 Sequencing System (Illumina, San Diego, CA, United States). High quality clean reads were obtained by filtering with Cutadapt (version 1.9) (https://cutadapt.readthedocs.io/en/stable/) and verified with FastQC (http://www.bioinformatics.babraham.ac.uk/projects/fastqc). Read alignment to the *Rattus norvegicus* reference genome was performed using HISAT2 package (version 2.0.4) (https://daehwankimlab.github.io/hisat2). Read assembly was performed using StringTie (version 1.3.4d) (http://ccb.jhu.edu/software/stringtie). StringTie and Ballgown (http://www.bioconductor.org/packages/release/bioc/html/ballgown.html) were used to estimate the relative expression levels (expressed as fragment per kilobase per million reads/FPKM values). In each experimental group, 3 independent samples were included. Gene expression data were analyzed with DESeq2 software (version 3.14) (https://bioconductor.org/packages/release/bioc/html/DESeq2.html). Genes with *p* value of < 0.05 and absolute fold change ≥ 2 were included for further bioinformatics analysis. The raw data set of RNA sequencing experiment was deposited at Dryad public repository (https://doi.org/10.5061/dryad.51c59zwb7).

### 2.5 Bioinformatics

Gene Ontology (GO) and Kyoto Encyclopedia of Genes and Genomes (KEGG) databases were used for gene functional annotations. Enrichment analysis was conducted using the ClusterProfiler package of R software (Yu et al., 2012), and gene clusters with false discovery rate (FDR) of < 0.05 were considered as significant.

### 2.6 Real-time PCR

Cells were homogenized in TRIzol Reagent (Thermo Fisher), and total RNA was isolated according to the manufacturer’s instructions. The RNA concentration was determined using a NanoDrop 2000 spectrophotometer (Thermo Fisher). cDNA was synthesized using PrimeScript RT-PCR Kit from TaKaRa (RR037A) (Otsu, Shiga, Japan). The PCR reaction was carried out using SYBR green qPCR mix (#CW0957M, from CoWin Biosciences, Taizhou, Jiangsu Province, China) on a StepOne Real-Time PCR System (Thermo Fisher). The primer sequences used in the study were listed in Supplementary Table S1.

### 2.7 Cell proliferation assay

Cells re-suspended in culture medium were seeded in 12-well plates (3 × 10^5^ cells per well). To inhibit p53, the cells were pretreated with pifithrin-α (20 μM) (#S5791 from Selleck Chemicals, Houston, TX, United States). After various treatments as indicated, the cells were pulse labeled with BrdU (10 μM) (from MedChemExpress, NJ, United States) for 1.5 h. BrdU incorporation was detected using immunofluorescence with a monoclonal BrdU antibody (#A20790, from ABclonal, Woburn, MA, United States) followed by Alexa Fluor 488-conjugated secondary antibody. The images were taken using a fluorescence microscope equipped with a DP74 CCD camera (Olympus, Tokyo, Japan).

### 2.8 Statistical analysis for individual genes

The numerical data of individual genes were analyzed with two-tailed unpaired *t*-test for comparison between two groups, or one-way ANOVA followed by Tukey’s test for multi comparisons, using Prism software (GraphPad, San Diego, CA, United States). A value of *p <* 0.05 was considered as significant.

## 3 Results

### 3.1 Transcriptome signatures induced by LPS and IFN-γ priming

**FIGURE 1:**
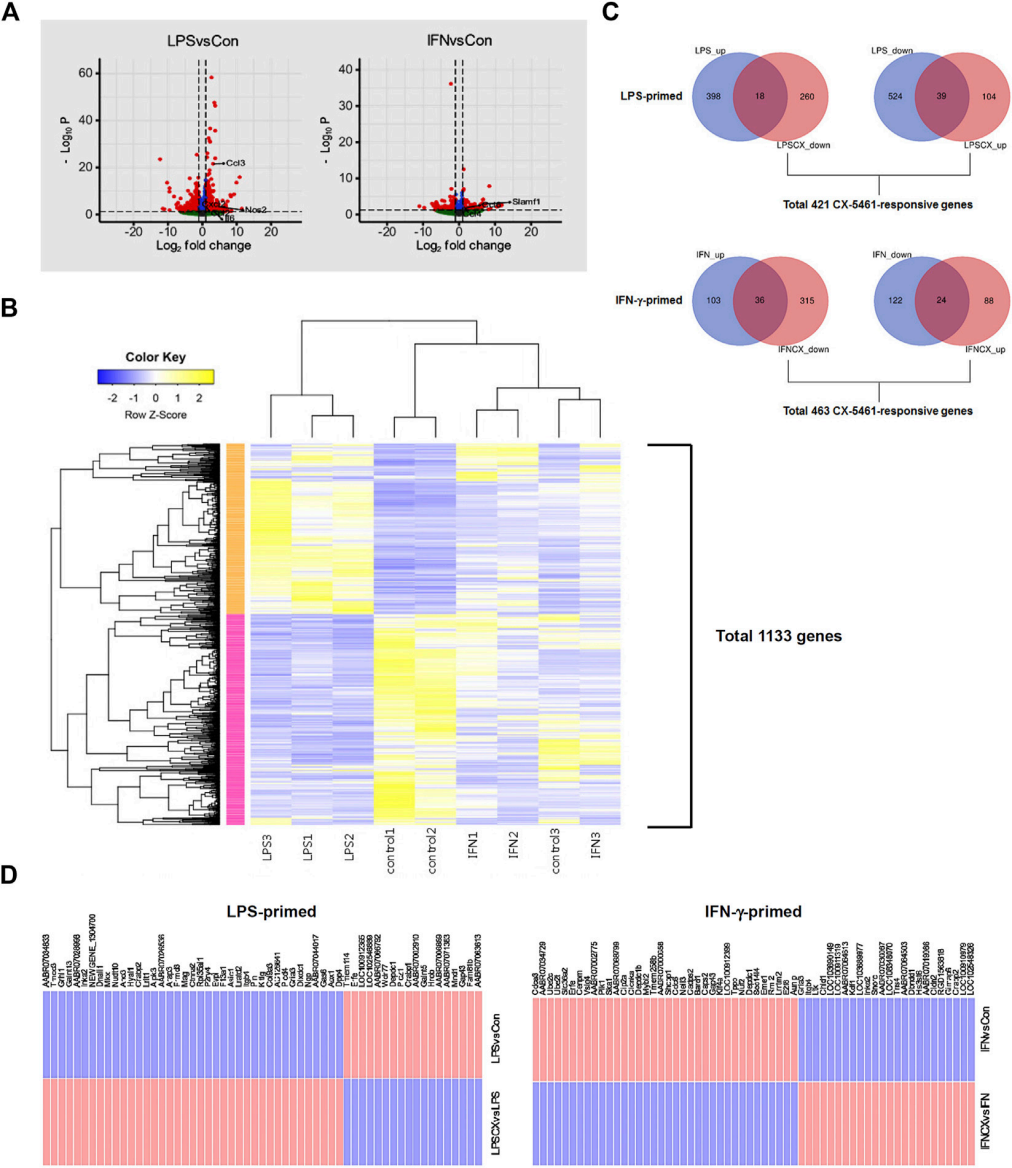
CX-5461 did not directly reverse the transcriptome signatures induced by LPS or IFN-γ in macrophages. **(A)** Volcano plots (log normalized *p* values versus log normalized fold change values) showing the genome-wide transcriptional effects of LPS and IFN-γ stimulation in primary macrophages. **(B)** Result of unsupervised k-means clustering analysis confirming that LPS and IFN-γ induced distinct transcriptome signatures in the macrophage (each treatment condition had 3 biological replications). **(C)** Venn diagrams showing that only a minor part of the transcriptome signature induced by LPS or IFN-γ was directly reversed by CX-5461 treatment. LPS_up: number of upregulated genes LPS treatment versus resting cells; LPSCX_down: downregulated genes LPS + CX-5461 versus LPS alone; LPS_down: downregulated genes LPS treatment versus resting cells; LPSCX_up: upregulated genes LPS + CX-5461 versus LPS alone. Grouping annotations were the same for IFN-γ-primed cells. The total numbers of CX-5461 responsive genes in LPS-primed and IFN-γ-primed cells were shown below the graphs respectively. **(D)** Lists of LPS- and IFN-γ-regulated genes which were directly reverted by CX-5461 treatment.

To delineate the potential anti-inflammatory activities of CX-5461, we first primed macrophage cells separately with LPS and IFN-γ, two classical pro-inflammatory stimuli with interweaving effects in macrophage (Schroder et al., 2006; Hu et al., 2008). LPS stimulation changed the expressions of 979 genes (Figure 1A; Supplementary Table S2), including several classical pro-inflammatory molecules such as *IL1A* (IL-1α), *IL1B* (IL-1β), *IL6* (IL-6), *NOS2* (inducible nitric oxide synthase/iNOS), *CCL2* (C-C motif chemokine ligand 2; aka. monocyte chemoattractant protein 1/MCP-1), *CCL3* (aka. macrophage inflammatory protein-1α/MIP-1α), *CXCL2* (C-X-C motif chemokine ligand 2; aka. MIP-2α), and *CCL12* (aka. MCP-5). IFN-γ stimulation changed the expressions of 285 genes (Figure 1A; Supplementary Table S3), including *CCL4* and *CCL6* (GO term: cellular response to interferon-gamma), which were moderately upregulated; and *SLAMF1* (signaling lymphocytic activation molecule family member 1) (GO term: negative regulation of interferon-gamma production), which was highly upregulated. The upregulation of *SLAMF1* gene in IFN-γ-primed macrophages was consistent with previous reports (Howie et al., 2002). Unsupervised k-means clustering analysis confirmed that LPS and IFN-γ induced distinct transcriptome signatures in the macrophage (Figure 1B).

### 3.2 CX-5461 did not directly reverse the transcriptome profiles associated with LPS or IFN-γ stimulation

According to the observations from our previous studies (Ye et al., 2017; Dai et al., 2018; Pang et al., 2021; Xu et al., 2021; Pan et al., 2022), activation of the p53 pathway following Pol I inhibition (rather than reduced productions of rRNAs and ribosomes) appears to have a predominant role in mediating CX-5461 effects. This phenomenon has also been recognized in cancer cells (Ferreira et al., 2020). Hence we first confirmed that CX-5461 at 1 μM (as established by our previous studies) effectively reduced the pre-rRNA level and stimulated p53 phosphorylation (on Ser15) under the present experimental settings (see Supplementary Figure S1). Using this concentration of CX-5461, we showed that of the 416 genes which were upregulated in LPS-primed macrophages (increased in LPS group as compared to control group), 18 were reverted by CX-5461 treatment (i.e., decreased in CX + LPS group as compared to LPS alone group); of the 563 genes which were downregulated in LPS-primed cells, 39 were reverted by CX-5461 (Figures 1C,D). Of the 139 genes which were upregulated in IFN-γ-primed cells, 36 were reverted by CX-5461; of the 146 genes which were downregulated in IFN-γ-primed cells, 24 were reverted by CX-5461 (Figures 1C,D; Supplementary Table S4). Overall, CX-5461 treatment reverted 6% of the genes which were changed by LPS, and 21% of those changed by IFN-γ. A special family of cytokines known as ELR^+^ CXCL cytokines (so named for their *N*-terminal glutamate, leucine, and arginine tripeptide motif preceding the C-X-C chemokine motif) have been shown to have important pro-inflammatory and pro-angiogenic effects (Giuliano et al., 2014), including *CXCL1, CXCL2*, *CXCL3, CXCL5, CXCL6, CXCL7* and *CXCL8*. Our study showed that, however, none of these ELR^+^ CXCL cytokines or their cognate receptors were significantly changed by CX-5461 treatment in macrophages.

### 3.3 CX-5461 treatment resulted in overlapping transcriptome signatures in LPS- and IFN-γ -primed macrophages

**FIGURE 2:**
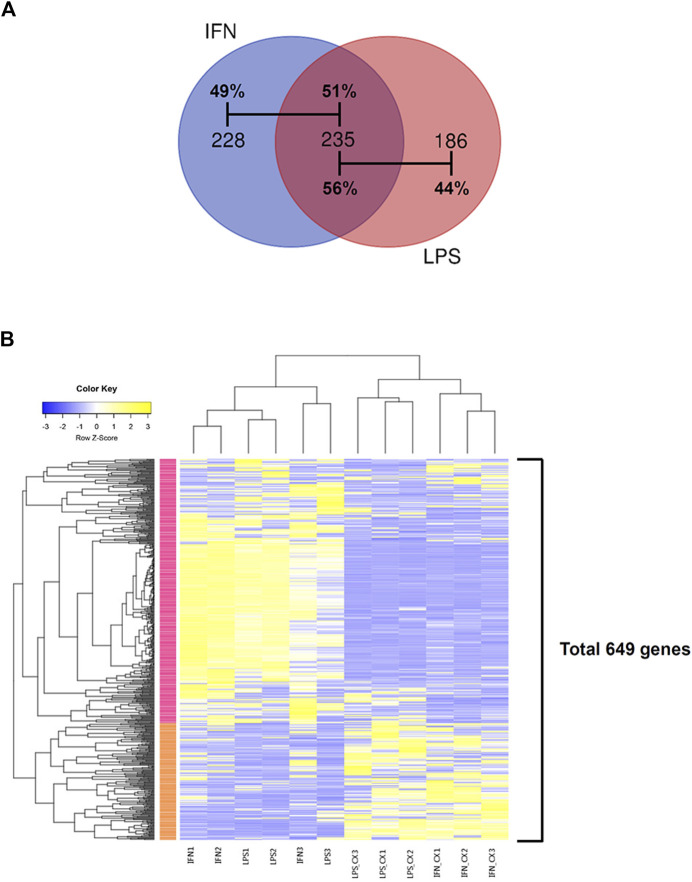
CX-5461 treatment resulted in overlapping transcriptome signatures in LPS- and IFN-γ-primed macrophages. **(A)** Venn diagrams showing that over 50% of the differentially regulated genes by CX-5461 were identical between LPS-primed and IFN-γ-primed cells. **(B)** Result of k-means clustering analysis confirming that CX-5461 treatment resulted in overlapping transcriptome signatures in LPS- and IFN-γ-primed macrophages.

**FIGURE 3:**
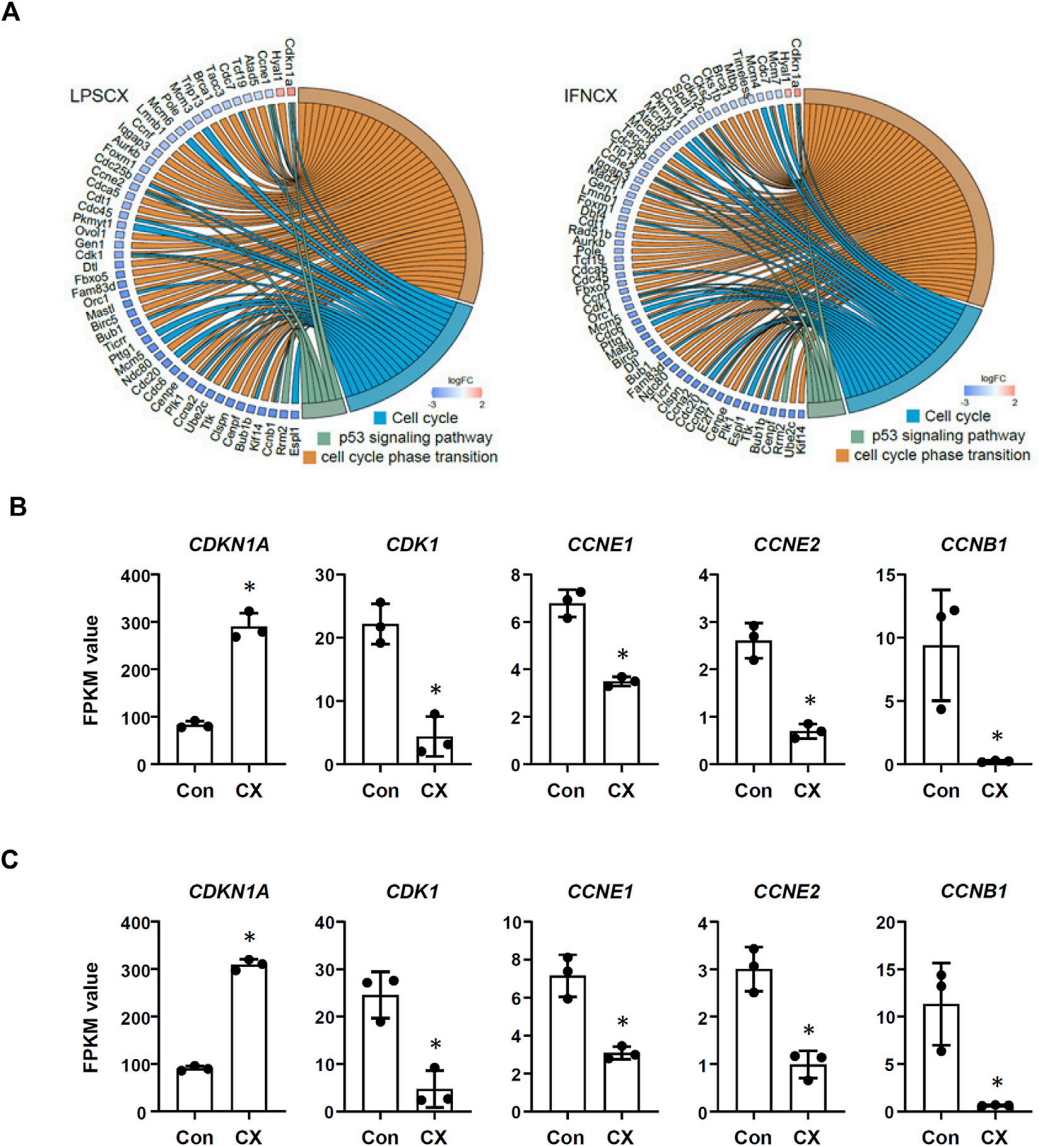
CX-5461 induced a molecular signature associated with cell cycle inhibition in macrophages. **(A)** Circos plots showing that CX-5461 regulated a cluster of genes which were related to cell cycle regulation in LPS- and IFN-γ-primed macrophages as identified by functional enrichment analyses. Some of these genes were linked to p53 signaling. **(B,C)** Numerical analysis of the expression levels of various genes involved in cell cycle regulation in LPS- and IFN-γ-primed macrophages respectively. The relative expression levels were expressed as fragment per kilobase per million reads (FPKM) values. CX, CX-5461. Bars represented mean ± standard deviation. *p < 0.05 versus vehicle control (Con), unpaired *t*-test.

In LPS-primed cells, CX-5461 caused changes in expression of 421 genes; in IFN-γ-primed cells, CX-5461 changed the expression of 463 genes (see Figure 1C, Figure 2A; Supplementary Table S5, S6). It is noted that of these differentially expressed genes, 235 genes were identical under the two experimental settings (account for 56% in LPS-primed cells and 51% in IFN-γ-primed cells) (Figure 2A). K-means clustering analysis confirmed that CX-5461 treatment resulted in overlapping transcriptome signatures in LPS- and IFN-γ-primed macrophages (Figure 2B). GO and KEGG analyses on the CX-5461-responsive genes similarly identified enrichment in biological processes related to cell cycle regulation (GO: cell cycle phase transition; KEGG: cell cycle), which were highly statistically significant (Table 1). Consistent with previous data suggesting that activation of the p53 pathway had a pivotal role in mediating the pharmacological effects of CX-5461 (Bywater et al., 2012; Tsai and Pederson, 2014; Ye et al., 2017; Cui et al., 2021; Pang et al., 2021; Xu et al., 2021), we also showed that a cluster of 6 genes associated with p53 signaling (KEGG: p53 signaling pathway) were significantly altered by CX-5461 in both LPS- and IFN-γ-primed cells. These p53-related genes included *CDKN1A* (cyclin-dependent kinase inhibitor 1/p21^Cip1^), *CDK1* (cyclin-dependent kinase 1/Cdk1), *CCNE2* (cyclin E2), *CCNE1* (cyclin E1), *RRM2* (ribonucleotide reductase regulatory subunit M2) and *CCNB*1 (cyclin B1). Except for *RRM2*, these genes were also present in the functional clusters of “cell cycle phase transition” and “cell cycle” as mentioned above (Figure 3A). Because *CDKN1A*, *CDK1*, *CCNE2*, *CCNE1* and *CCNB*1 were all key regulators of cell cycle, we plotted the numerical expression data for these genes, and demonstrated that CX-5461 upregulated *CDKN1A* (cell cycle inhibitor) and downregulated *CDK1*, *CCNE2*, *CCNE1* and *CCNB1* (mediators of cell cycle) in both LPS- and IFN-γ-primed cells (Figures 3B,C), supporting that CX-5461 predominantly inhibited cell cycle progression in macrophages. Because the group size (*n* = 3 each) in the sequencing experiment was relatively small (restricted by the total cost), we further confirmed the key findings shown in Figures 3B,C using real-time PCR assays. We demonstrated that CX-5461 treatment significantly upregulated *CDKN1A*, and downregulated *CDK1*, *CCNE2*, *CCNE1* and *CCNB1*, in both LPS- and IFN-γ-primed cells (Supplementary Figure S2).

**TABLE 1:** Functional enrichment analysis showing that CX-5461 changed the expression of a cluster of genes associated with cell cycle regulation in macrophages.

	LPS-primed		IFN-γ-primed	
***GO/KEGG terms***	GO: cell cycle phase transition	KEGG: cell cycle	GO: cell cycle phase transition	KEGG: cell cycle
***Gene ratio (adjusted*** *p* ***value)***	45/335 (8.41 × 10^–24^)	23/126 (1.39 × 10^–17^)	52/363 (3.17 × 10^–29^)	27/138 (8.26 × 10^–22^)
***Specific genes***	Atad5/Aurkb/Birc5/Brca1/Bub1/Bub1b/Ccna2/Ccnb1/Ccne1/Ccne2/Ccnf/Cdc25b/Cdc45/Cdc6/Cdc7/Cdca5/Cdk1/Cdkn1a/Cdt1/Cenpe/Cenpf/Clspn/Dtl/E2f7/Fam83d/Fbxo5/Foxm1/Gen1/Hyal1/Iqgap3/Kif14/Lmnb1/Mad2l1/Mastl/Ndc80/Orc1/Ovol1/Plk1/Pole/Tacc3/Tcf19/Ticrr/Trip13/Ttk/Ube2c	Bub1/Bub1b/Ccna2/Ccnb1/Ccne1/Ccne2/Cdc20/Cdc25b/Cdc45/Cdc6/Cdc7/Cdk1/Cdkn1a/Espl1/Mad2l1/Mcm3/Mcm5/Mcm6/Orc1/Pkmyt1/Plk1/Pttg1/Ttk	Atad5/Aurkb/Birc5/Brca1/Bub1/Bub1b/Ccna2/Ccnb1/Ccne1/Ccne2/Ccnf/Cdc25b/Cdc45/Cdc6/Cdc7/Cdca5/Cdk1/Cdkn1a/Cdkn2c/Cdt1/Cenpe/Cenpf/Cks1b/Cks2/Clspn/Dbf4/Dtl/E2f7/Fam83d/Fbxo5/Foxm1/Gen1/Hyal1/Iqgap3/Kif14/Lmnb1/Mad2l1/Mastl/Mtbp/Ndc80/Orc1/Plk1/Pole/Rad51b/Spdl1/Tacc3/Tcf19/Ticrr/Timeless/Trip13/Ttk/Ube2c	Bub1/Bub1b/Ccna2/Ccnb1/Ccne1/Ccne2/Cdc20/Cdc25b/Cdc45/Cdc6/Cdc7/Cdk1/Cdkn1a/Cdkn2c/Dbf4/Espl1/Mad2l1/Mcm3/Mcm4/Mcm5/Mcm6/Mcm7/Orc1/Pkmyt1/Plk1/Pttg1/Ttk

### 3.4 CX-5461 treatment and DNA repair pathways

Previous results from our and other groups suggested that induction of DDR featuring ATM/ATR pathway activation was a prominent characteristic of the CX-5461 actions in tumor cell lines and in cardiovascular cells (Negi and Brown, 2015; Quin et al., 2016; Ye et al., 2017; Pang et al., 2021). Hence we examined whether CX-5461 treatment in macrophages was transcriptionally linked to DDR pathways. We found that in both LPS- and IFN-γ-primed macrophages, CX-5461 altered the expression pattern of a gene cluster associated with DDR (GO: DNA repair) (see Table 2). This functional cluster included 45 genes in LPS-primed cells and 56 in IFN-γ-primed cells, of which the majority (41 genes) were identical between the two groups. Among these CX-5461-responsive genes, there were multiple well-established factors involved in double-strand break repair/homologous recombination, such as *RAD51, BRCA1* (breast cancer type 1 susceptibility protein), *RAD51AP1* (Rad51 associated protein 1), and *GEN1* (GEN1 Holliday Junction 5′ flap endonuclease). CX-5461 also changed several genes which were essential for DNA replication, such as *CDC45* (cell division cycle 45) and *MCM* (minichromosome maintenance complex component) members 3, 5 and 6. However, further quantitative analysis demonstrated that CX-5461 consistently downregulated the expression of these genes in macrophage (see Supplementary Table S5, S6).

**TABLE 2:** Functional enrichment analysis showing that CX-5461 changed the expression of a cluster of genes associated with DNA damage response in macrophages.

	LPS-primed	IFN-γ-primed
**GO terms**	DNA repair	DNA repair
**Gene ratio (adjusted** p **value)**	45/335 (2.24 × 10^–21^)	56/363 (7.39 × 10^–30^)
**Specific genes**	Aunip/Bard1/Brca1/Brip1/Cdc45/Cdc7/Cdca5/Chaf1a/Chaf1b/Clspn/Ddx11/Dtl/Eme1/Esco2/Exo1/Fanca/Fancb/Fancc/Fancd2/Fanci/Foxm1/Gen1/Hrob/Kif22/Mcm3/Mcm5/Mcm6/Mcm8/Mms22l/Neil3/Parpbp/Pclaf/Pif1/Pola1/Pold1/Pole/Polq/Pttg1/Rad51/Rad51ap1/Rad54l/Ticrr/Trip13/Ube2t/Uhrf1	Aunip/Bard1/Brca1/Brip1/Cdc45/Cdc7/Cdca5/Chaf1a/Chaf1b/Clspn/Ddx11/Dtl/Eme1/Esco2/Exo1/Fanca/Fancb/Fancd2/Fanci/Fignl1/Foxm1/Gen1/Gins4/H2ax/Hmga2/Hrob/Kif22/Mcm3/Mcm4/Mcm5/Mcm6/Mcm7/Mms22l/Neil3/Parpbp/Pclaf/Pif1/Pola1/Pole/Pole2/Prmt6/Pttg1/Rad18/Rad51/Rad51ap1/Rad51b/Rad54b/Rad54l/Rmi2/Ticrr/Timeless/Tonsl/Trip13/Ube2t/Uhrf1/Xrcc2

Underlined genes were those identical between LPS-primed and IFN-γ-primed cells.

### 3.5 Effects of CX-5461 treatment on individual inflammatory genes

**FIGURE 4:**
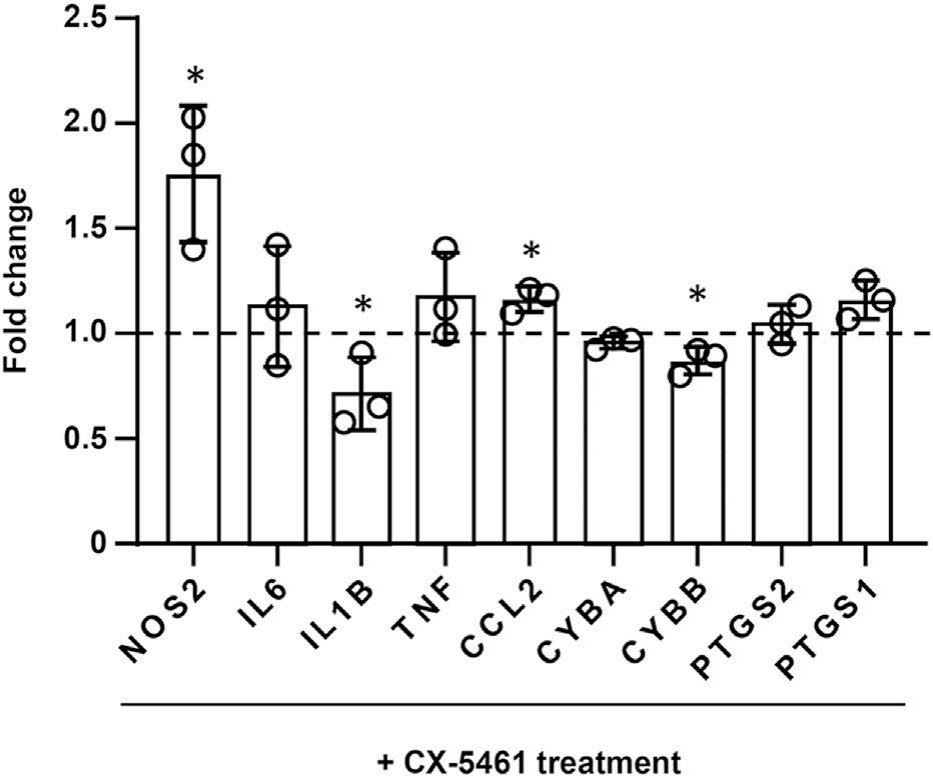
Effects of CX-5461 treatment on the expression of individual inflammatory genes in LPS-primed macrophages. FPKM values of CX-5461-treated cells were normalized to corresponding control cells and expressed as fold of control. Bars represented mean ± standard deviation. *p < 0.05 versus control, unpaired *t*-test.

As mentioned above, treatment with CX-5461 did not induce a distinct anti-inflammatory transcriptional signature in LPS- or IFN-γ-primed macrophages. Therefore, we examined the changes of several individual genes with critical pro-inflammatory functions in the macrophage, including *NOS2*, *IL6*, *IL1B*, *TNF* (TNF-α), *CCL2*, and *PTGS1* (prostaglandin-endoperoxide synthase 1; aka. cyclooxygenase-1/COX-1) and *PTGS2* (aka COX-2), in LPS-primed cells. It was found that the effects of CX-5461 on these genes were variable (Figure 4); only the expression of *IL1B* was decreased by CX-5461 (*p* = 0.0462). In addition, we examined the effects of CX-5461 on *CYBA* (cytochrome b-245 α chain; aka. p22phox) and *CYBB* (cytochrome b-245 β chain; aka. gp91phox), two membrane-bound subunits of the phagocytic NADPH oxidase. CX-5461 decreased the expression of *CYBB* (*p* = 0.0262), but not *CYBA* (Figure 4). We further examined the effects of CX-5461 on the expression of these pro-inflammatory genes using real-time PCR. Confirming our genomics data, these experiments demonstrated variable effects of CX-5461 (see Supplementary Figure S3), supporting the notion that the anti-inflammatory action of CX-5461 in macrophages is unlikely to be attributable to a systemic repression of major pro-inflammatory genes.

### 3.6 Pifithrin-α blunted the inhibitory effect of CX-5461 on macrophage proliferation

To further confirm that p53 activation was functionally involved in the inhibitory effect of CX-5461 on macrophage proliferation, we pretreated the cells with the selective p53 inhibitor pifithrin-α. We demonstrated that pifithrin-α blunted the inhibitory effect of CX-5461 on macrophage proliferation (Supplementary Figure S4).

## 4 Discussion

In the present study, we attempt to delineate the molecular mechanisms underlying the observed anti-inflammatory effects of CX-5461 in macrophages, by characterizing the transcriptome signatures in response to CX-5461 treatment. The most consistent finding from our study is that in both LPS- and IFN-γ-primed macrophages, CX-5461 predominantly triggers a transcriptional program favoring cell cycle inhibition, featuring downregulations of genes encoding cell cycle mediators and concomitant upregulations of cell cycle inhibitors. On the other hand, our data do not support that, under the present experimental settings, CX-5461 can directly induce a distinct anti-inflammatory transcriptional program in macrophages. Therefore, it is suggested that the most important mechanism by which CX-5461 regulates macrophage-mediated inflammation is to modulate the expansion of the macrophage cell population. This conclusion is consistent with our previous observation that CX-5461 can significantly inhibit macrophage proliferation both *in vitro* and *in vivo* (Dai et al., 2018). Moreover, our bioinformatics results further corroborate the central role of p53 in mediating the cell cycle regulatory effect of CX-5461 in macrophages.

It is well recognized that chemotactic migration of monocytes from circulation to the inflamed tissue, and *in situ* differentiation of the recruited monocytes to mature macrophages, constitute the major pathway leading to accumulation of inflammatory macrophages and release of pro-inflammatory cytokines at the site of inflammation (Haskill et al., 1992; Ohashi et al., 2015). However, emerging evidence suggests that in addition to the mobilization of monocytes/macrophages, localized macrophage proliferation may also have a crucial contribution to the maintenance of inflammatory reaction. This mechanism may be of particular relevance in the chronic inflammatory conditions associated with blood vessels. For example, multiple studies have shown that macrophage proliferation facilitated by the local micro-environment in the vessel wall is a predominant contributor to the development of atherosclerosis (Robbins et al., 2013; Tang et al., 2015; Tanaka et al., 2016). This notion is also supported by our previous results indicating that inhibiting macrophage proliferation by CX-5461 may at least partly contribute to the amelioration of aortic transplantation-induced vascular inflammation and remodeling (Dai et al., 2018). Apart from vascular inflammation, there is evidence showing that local macrophage proliferation is also important for parasite-induced Th2 type inflammation (Jenkins et al., 2011).

Independent lines of evidence suggest that induction of ATM/ATR-mediated DDR is a prominent upstream signaling event of CX-5461-induced cellular effects, which may result in p53-dependent or p53-independent cytostatic actions in proliferating cells (Negi and Brown, 2015; Quin et al., 2016; Ye et al., 2017; Sanij et al., 2020; Pang et al., 2021). In the present study, we have shown that CX-5461 treatment in macrophages is indeed associated with a transcriptome signature related to DNA repair. Specifically, CX-5461 downregulates the expression of multiple genes involved in homologous recombination, an important pathway responsible for repairing double-strand breaks and maintaining genome stability (Spies et al., 2021). These data seem to be paradoxical to the observed activation of DDR in CX-5461-treated cells, and the physiological significance of CX-5461-induced downregulations of the DDR genes remain elusive. Nevertheless, our data are exactly consistent with previous studies in which p53-dependent downregulations of *RAD51, BRCA1* and *MCM5* expression have been reported (Arizti et al., 2000; Arias-Lopez et al., 2006; Agarwal et al., 2007), further highlighting p53 in orchestrating the molecular networks that are responsive to CX-5461 treatment.

Although the available data regarding the role of p53 in modulating inflammatory reactions appear to be controversial, a mass of evidence suggests that p53 has anti-inflammatory activities in certain pathological contexts (see Cui et al., 2021). In a recent study we have shown that CX-5461 can inhibit T cell-mediated immunity functions *via* p53-DUSP5 (dual-specificity phosphatase 5) axis and subsequent antagonism of the Erk1/2 mitogen-activated protein kinase activity, thereby preventing the occurrence of acute transplant rejection (Pan et al., 2022). These results suggest that CX-5461 has independent modulatory activities on inflammatory/immune signalings in immune cells apart from regulation of the cell cycle. Supporting this notion, different studies have demonstrated negative correlations between p53 and the expression of prototypic inflammatory genes including *IL6* (Santhanam et al., 1991), *IL1B* (Yamanishi et al., 2002), *NOS2* (Huang et al., 2014) and *TNF* (Tang et al., 2011). Our present genomic data also confirm that CX-5461 downregulates expressions of IL-1β and gp91phox (the catalytic subunit of superoxide-producing NADPH oxidase). However, the effects of CX-5461 on the expression of other inflammatory genes appear to be variable; the relatively weak effects of CX-5461 on macrophage inflammatory gene expression in comparison to our previous data might be due to the lower concentration of CX-5461 used in the present study.

In conclusion, CX-5461 principally induces a molecular signature related to cell cycle inhibition in macrophages, featuring downregulation of genes encoding cell cycle mediators and concomitant upregulation of cell cycle inhibitors. On the other hand, at a concentration which is effective for regulating macrophage proliferation, CX-5461 does not necessarily induce a distinct anti-inflammatory transcriptional program, suggesting that limiting cell proliferation predominates in the inhibitory effects of CX-5461 on macrophage-mediated inflammation.

## Data Availability

The datasets presented in this study can be found in online repositories. The names of the repository/repositories and accession number(s) can be found below: https://datadryad.org/stash/dataset/doi:10.5061/dryad.51c59zwb7.
