# Supplementary material for: A transcriptional program associated with cell cycle regulation predominates in the anti-inflammatory effects of CX-5461 in macrophage
Source: Front Pharmacol. 2022 Oct 26;13:926317. doi: 10.3389/fphar.2022.926317 (PMC9644203; doi:10.3389/fphar.2022.926317)
Supplement: Supplementary file 3 [file DataSheet4.PDF]

Supplementary Figure S4

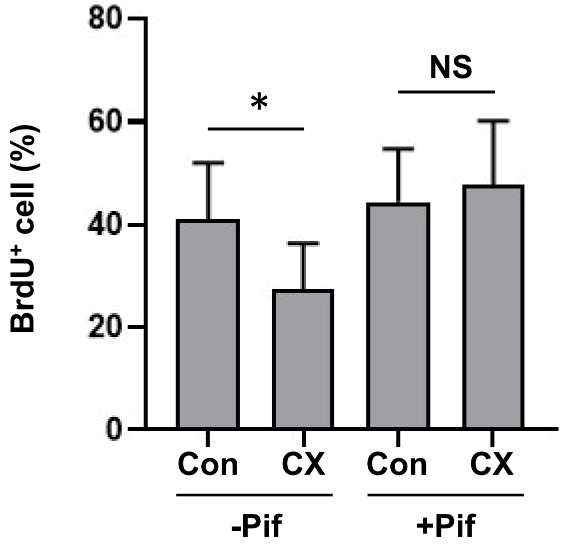

Figure S4. The p53 inhibitor pifithrin-α blunted the inhibitory effect of CX-5461 (CX) on cell proliferation measured with BrdU incorporation assay in LPS-primed macrophages. pifithrin-α (Pif) was used at 20 μM. Data were expressed as mean ± standard deviation. * $P < 0.05$, one-way ANOVA ($n = 6$ in each group). NS, no significance.
