# Supplementary material for: A transcriptional program associated with cell cycle regulation predominates in the anti-inflammatory effects of CX-5461 in macrophage
Source: Front Pharmacol. 2022 Oct 26;13:926317. doi: 10.3389/fphar.2022.926317 (PMC9644203; doi:10.3389/fphar.2022.926317)
Supplement: Supplementary file 5 [file Table1.DOCX]

**Supplementary Table S1. Primer sequences used for real-time PCR assays**

|  | **Forward** | **Reverse** |
| --- | --- | --- |
| ***GAPDH*** | TCTCTGCTCCTCCCTGTTCT | ATCCGTTCACACCGACCTTC |
| ***18S mature rRNA*** | GTAACCCGTTGAACCCCATT | CCATCCAATCGGTAGTAGCG |
| ***45S pre-rRNA*** | GTTCCGCTCACACCTCAGAT | AGTGCGTTCGAAGTGTCGAT |
| ***CDKN1A*** | CCCGAGAACGGTGGAACTTT | GAACACGCTCCCAGACGTAG |
| ***CDK1*** | CTTTTCCACGGCGACTCAGA | GCAAGTCCAAGCCGTTTTCA |
| ***CCNE1*** | AAGAAGAAGGTGGCTCCGAC | GGGATGAAAGAGCAGGGGTC |
| ***CCNE2*** | ATCTACGCTCCCAAGCTCCA | ACTGTTACTGGACAAAGTTCCC |
| ***CCNB1*** | AACCCCTGCTGAGATCGAGA | CCACAGGTTTTGGTAGGGCT |
| ***NOS2*** | GGAGAAAACCCCAGGTGCTA | GTGAGGAACTGGGGGAAACC |
| ***IL1B*** | GGGATGATGACGACCTGCTA | ACAGCACGAGGCATTTTTGT |
| ***IL6*** | TTTCTCTCCGCAAGAGACTTCC | TGTGGGTGGTATCCTCTGTGA |
| ***TNF*** | ATGGGCTCCCTCTCATCAGT | GCTTGGTGGTTTGCTACGAC |
| ***CCL2*** | TGATCCCAATGAGTCGGCTG | GGTGCTGAAGTCCTTAGGGT |
| ***CYBA*** | TTGCAGGAGTGCTCATCTGT | GGTACTTCTGTCCACACCGC |
| ***CYBB*** | CATTTTCGTCAAGCGTCCCG | AGTCGCCAACAATGCGGATA |
| ***PTGS2*** | GATGACGAGCGACTGTTCCA | TGGTAACCGCTCAGGTGTTG |
| ***PTGS1*** | GAAACCCAGCACATTCGGTG | TCTTGGTGTTGAGGCAGACC |
