# Supplementary material for: A transcriptional program associated with cell cycle regulation predominates in the anti-inflammatory effects of CX-5461 in macrophage
Source: Front Pharmacol. 2022 Oct 26;13:926317. doi: 10.3389/fphar.2022.926317 (PMC9644203; doi:10.3389/fphar.2022.926317)
Supplement: Supplementary file 6 [file DataSheet3.PDF]

Supplementary Figure S3

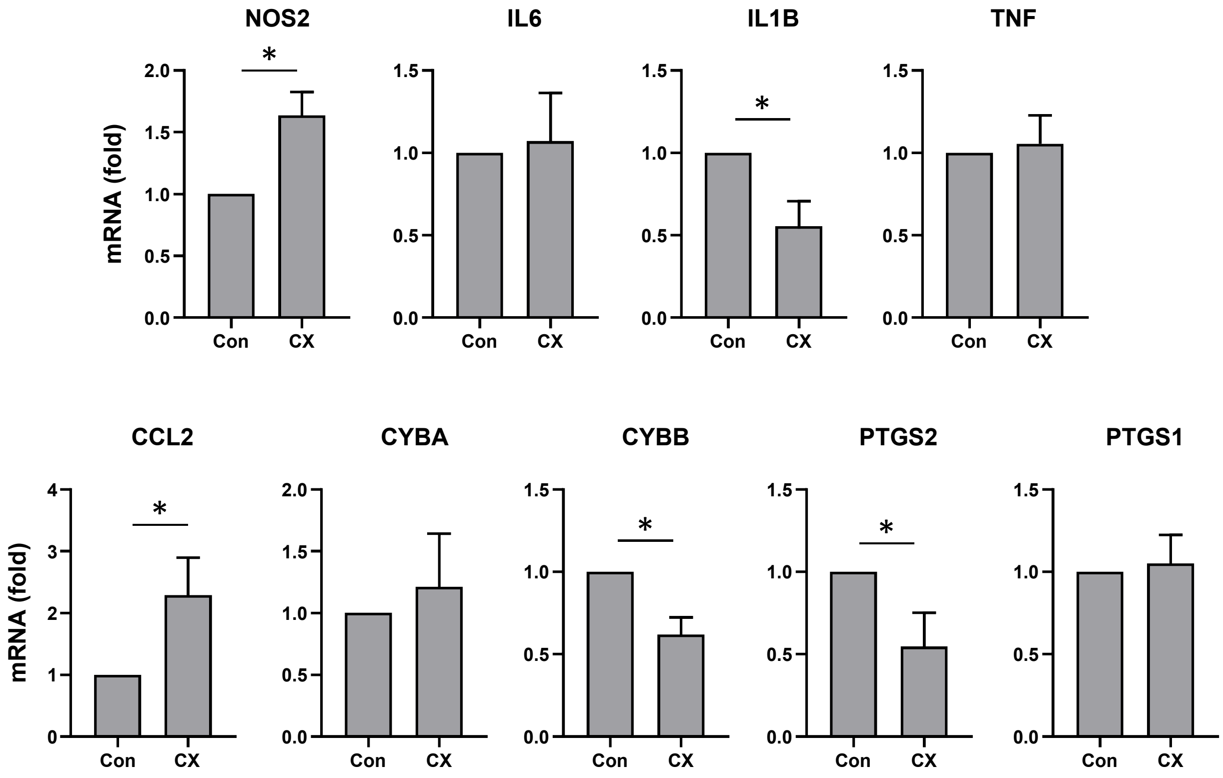

Figure S3. Real-time PCR results showing that CX-5461 (CX) treatment had variable effects on the expression of several pro-inflammatory genes in LPS-primed macrophages. Data were expressed as mean ± standard deviation. * $P < 0.05$, unpaired *t*-test ($n = 6$ in each group).
