# Supplementary material for: A transcriptional program associated with cell cycle regulation predominates in the anti-inflammatory effects of CX-5461 in macrophage
Source: Front Pharmacol. 2022 Oct 26;13:926317. doi: 10.3389/fphar.2022.926317 (PMC9644203; doi:10.3389/fphar.2022.926317)
Supplement: Supplementary file 8 [file DataSheet1.PDF]

Supplementary Figure S1

(A)

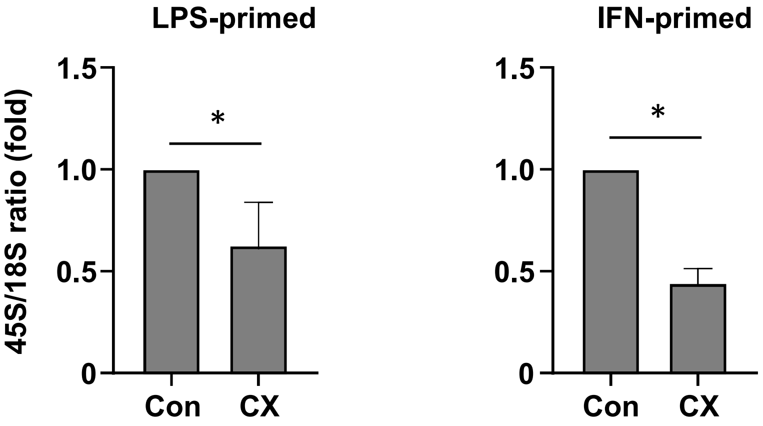

(B)

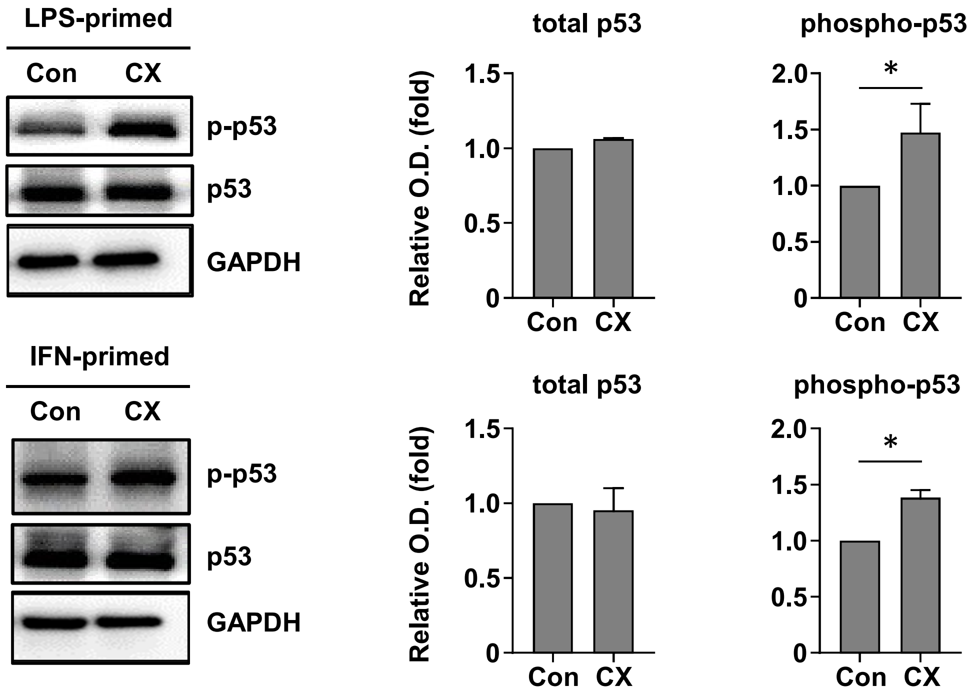

Figure S1. Real-time PCR (A) and western blot (B) results showing that CX-5461 (CX) at 1 μM effectively reduced the relative 45S pre-rRNA level and stimulated p53 phosphorylation (Ser15) in LPS-primed and IFN-γ-primed primary macrophage cells. Data were expressed as mean ± standard deviation. * *P* < 0.05, one-way ANOVA (*n* = 4 in each group).
